# Ovarian Cancer Pathogenesis: A Model in Evolution

**DOI:** 10.1155/2010/932371

**Published:** 2009-09-06

**Authors:** Alison M. Karst, Ronny Drapkin

**Affiliations:** ^1^Department of Medical Oncology, Center for Molecular Oncologic Pathology, Dana-Farber Cancer Institute, 44 Binney Street, Boston, MA 02115, USA; ^2^Department of Pathology, Brigham and Women's Hospital, 75 Francis Street, Boston, MA 02115, USA

## Abstract

Ovarian cancer is a deadly disease for which there is no effective means of early detection. Ovarian carcinomas comprise a diverse group of neoplasms, exhibiting a wide range of morphological characteristics, clinical manifestations, genetic alterations, and tumor behaviors. This high degree of heterogeneity presents a major clinical challenge in both diagnosing and treating ovarian cancer. Furthermore, the early events leading to ovarian carcinoma development are poorly understood, thus complicating efforts to develop screening modalities for this disease. Here, we provide an overview of the current models of ovarian cancer pathogenesis, highlighting recent findings implicating the fallopian tube fimbria as a possible site of origin of ovarian carcinomas. The ovarian cancer model will continue to evolve as we learn more about the genetics and etiology of this disease.

## 1. Introduction

Ovarian cancer afflicts ~204 000 women worldwide each year, including ~21 650 Americans [1, 2]. Despite its relatively low incidence rate, ovarian cancer is an extremely lethal disease. Globally, it claims 125 000 lives per year, making it the seventh leading cause of cancer-related deaths among women [2]. In the United States, ovarian cancer mortality is even higher; it ranks as the fifth deadliest malignancy among women, with an estimated 15 520 deaths per year [1]. In fact, of the top ten cancer types afflicting American women in 2008, ovarian cancer had the highest death-to-incidence ratio, exceeding even that of lung cancer [1]. Its high mortality is primarily due to difficulties in diagnosing early stage disease. Although the 5 years survival rate for stage I ovarian cancer is >90%, stage I diagnoses are more often the exception than the rule. Most patients (*∼*75%) present with advanced stage (III/IV) tumors, for which the 5 years survival rate is a dismal 30% [1]. This is not surprising when one considers the anatomical problem—the ovaries are a pair of tiny organs, only ~2–4 cm in diameter, suspended on either side of the uterus and not readily accessible by pelvic examination unless significantly enlarged. By definition, a stage I tumor is confined to the ovary [3] and is therefore unlikely to be noticed without the aid of a sensitive screening test. Unfortunately, there are currently no effective screening modalities for detecting ovarian cancer in asymptomatic individuals. Furthermore, there are no tell-tale physical signs of the disease. Typical symptoms—which include abdominal discomfort, bloating, gas, nausea, and urinary urgency—are vague and often mistaken for gastrointestinal problems [4, 5]. In many cases, symptoms may not even present until the tumor has reached an advanced stage. Consequently, ovarian cancer is frequently nicknamed the “silent killer” [5–7].

## 2. Early Detection

The best tools currently available for detecting early-stage ovarian cancer are transvaginal sonography (TVS) and serum biomarker testing. TVS is a noninvasive technique that provides detailed images of ovary size and shape, facilitating the detection of ovarian masses. Large-scale studies evaluating its ability to identify early-stage tumors have reported mixed results [8–13]. Overall, TVS has not demonstrated adequate sensitivity to warrant its use in screening general populations. Furthermore, even in the most optimistic reports, it is clear that TVS can only detect tumors that cause a significant increase in ovarian volume [13]. This is especially worrisome in the case of serous-type tumors which may spread rapidly from the ovary to other pelvic sites prior to ovarian enlargement. For instance, in a large study by van Nagell et al., four women were diagnosed with late-stage ovarian cancer within 12 months of a negative TVS scan [9]. In addition, there is debate as to whether all ovarian tumors actually arise from the ovary and not from adjacent pelvic sites such as the fallopian tube or peritoneum [14]. Serous tumors involving the ovary but originating from extraovarian sites can never be classified as “stage I” according to current staging systems, since they are never confined to the ovary. It has therefore been proposed that the definition of “early stage” ovarian carcinoma should be based on minimal tumor volume rather than anatomical location [14]. TVS screening may also be prone to false-positive results because it cannot always distinguish malignant ovarian tumors from benign adnexal masses, such as cysts and fibromas, which are highly prevalent among postmenopausal women [15, 16]. While TVS alone is not a suitable front-line screening modality, it can serve as a useful secondary screening tool after serum biomarker testing [17, 18].

Serum biomarker testing is an ideal form of cancer detection because it is minimally invasive, cost effective, easily administered, nonsubjective, and is not contingent upon primary ovarian involvement. The most well-studied ovarian cancer biomarker is CA-125, a high molecular weight transmembrane glycoprotein expressed by coelomic- and Müllerian-derived epithelia, including that of the fallopian tube, endometrium, and endocervix [19]. It is not expressed by normal ovarian epithelium [20]. CA-125 is detected at low levels (<35 U/mL) in the serum of healthy individuals but is elevated in ~50% of stage I ovarian cancer patients and ~90% of advanced-stage patients [21–24]. CA-125 elevation is predominantly associated with serous tumors, the most common and most lethal subtype of ovarian carcinoma [25]. Serum levels directly correlate with the level of CA-125 protein production in tumor cells and appear to reflect a state of active tumor growth [25–27]. Following its discovery in 1981 [28], CA-125 was intensely studied to evaluate its potential for detecting early-stage ovarian cancer, and many encouraging results were reported. For example, a study of prediagnostic serum samples found that CA-125 was elevated in 25% of ovarian cancer patients 5 years prior to their diagnoses [29]. However, it was later discovered that serum CA-125 levels can be increased by a range of benign conditions (such as pelvic inflammatory disease, endometriosis, uterine fibroids, and ovarian cysts) making false positivity a problem [30]. So far, CA-125 has not demonstrated adequate sensitivity to support its use in screening asymptomatic women for early-stage ovarian cancer [31], but longer-term studies are still underway. In the meantime, CA-125 remains a valuable tool for monitoring response to chemotherapy and for detecting disease relapse following treatment [32, 33]. 

In an attempt to improve sensitivity for early disease detection, two approaches have been taken. (1) Obtaining longitudinal measurements of CA-125, and (2) using multiple tumor markers. The former approach assumes that CA-125 levels are likely to remain stable in patients with benign conditions but will increase over time if an ovarian cancer is in progress. Thus, by plotting serial CA-125 measurements over a period of years, one can calculate the “probability of ovarian cancer” for an individual patient using a Bayesian algorithm [34–36]. 

The latter approach aims to combine CA-125 with one or more additional tumor markers, most notably human epididymis protein 4 (HE4). HE4 is a glycoprotein secreted by Müllerian epithelia of the female reproductive tract as well as male epididymis [37]. Like CA-125, it is not expressed by normal ovarian epithelium but appears in some premalignant ovarian cysts (cortical inclusion cysts) and is strongly expressed by the most common ovarian tumor subtypes (serous and endometrioid) [37]. HE4 is both more sensitive and specific than CA-125 in detecting early-stage ovarian cancer and is not associated with benign conditions to the same degree, enabling HE4 to distinguish malignant ovarian tumors from benign cystic lesions [38–40]. When used in combination to detect early-stage disease, CA-125 and HE4 perform better than either marker alone and can be used to stratify patients into high- and low-risk groups [40, 41]. Although HE4 was recently approved by the US Food and Drug Administration for use in monitoring ovarian cancer patients following treatment, larger-scale studies are required to determine whether a dual CA-125/HE4 biomarker test is suitable for screening asymptomatic women in the general population. 

In addition, some groups have assembled panels of biomarkers to create a so-called “composite marker.” For example, Zhang et al. measured the serum levels of four tumor markers (CA-125II, CA72-4, CA15-3, and M-CSF) in sets of healthy women, women with benign ovarian conditions, and women with ovarian cancer [42]. From their measurements, they derived an artificial neural network (ANN) model that could distinguish healthy women from those with early-stage ovarian cancer. The sensitivity of their ANN-derived composite index was 25–28% higher than that of CA-125 alone. Similar reports of increased sensitivity upon employment of multiple tumor markers have been reported by other groups [43, 44]. However, serum tests for multiple biomarkers are not yet widely available.

## 3. Ovarian Cancer Is a Heterogeneous Disease

**Figure 1:**
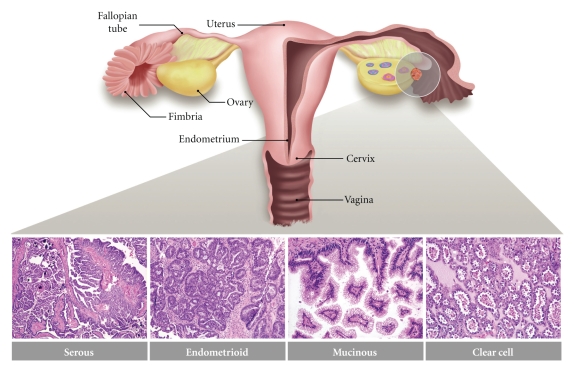
The major histologic subtypes of ovarian carcinoma. Serous carcinomas resemble fallopian tube epithelium, endometrioid carcinomas resemble endometrial glands, and mucinous carcinomas resemble endocervical epithelium. Photographs show representative tumor sections stained with hematoxylin and eosin. The shaded circle represents the general anatomical location from which ovarian carcinomas are thought to arise. The pink and blue entities within the cross-sected ovary represent maturing ovarian follicles.

Some of the greatest challenges in detecting and treating ovarian cancer stem from its heterogeneous nature. The term “ovarian cancer” refers not to a single disease, but to a diverse group of malignancies affecting the ovary. In general, ovarian tumors may develop from one of three cell types: epithelial cells, sex cord-stromal cells (including granulosa, theca, and hilus cells), or germ cells (oocytes). Although ~40% of all ovarian tumors are nonepithelial in origin, such lesions rarely progress to a malignant state and account for only 10% of ovarian cancers [45]. This paper will therefore focus exclusively on epithelial-derived ovarian tumors, which constitute the predominant and most lethal forms of the disease. Epithelial ovarian carcinomas are themselves a heterogeneous group of neoplasms that exhibit a wide range of tumor morphologies, clinical manifestations, and underlying genetic alterations. Upon diagnosis of malignancy, ovarian tumors are surgically staged to determine how far they have extended beyond the ovary [3]. Stage I indicates confinement to the ovary. Stage II tumors extend beyond the ovary to adjacent pelvic structures such as the fallopian tube or uterus. Stage III indicates metastasis to the peritoneum and/or regional lymph nodes. Stage IV tumors have metastasized beyond the peritoneum to distant sites. Additionally, tumors are classified by subtype. Current clinical guidelines set forth by the World Health Organization (WHO) recognize eight histologic tumor subtypes: serous, endometrioid, mucinous, clear cell, transitional cell, squamous cell, mixed epithelial, and undifferentiated [46]. The three most common—serous, endometrioid, and mucinous—are characterized by their morphological resemblance to various mucosal tissues of the female reproductive tract, all of which exhibit Müllerian differentiation (Figure 1). More specifically, serous tumors resemble fallopian tube epithelium, endometrioid tumors resemble endometrial glands, and mucinous tumors resemble endocervical epithelium (though clinically, distinguishing mucinous ovarian tumors from those of the gastrointestinal tract is more relevant) [47]. This phenomenon is quite remarkable when one considers that all ovarian tumor subtypes are conventionally thought to develop from ovarian surface epithelium, a monolayer of nondescript, poorly differentiated mesothelial cells [48]. Within each subtype, tumors are further described as either benign, malignant, or borderline and, depending upon tumor subtype, classified as low- or high-grade. Borderline tumors are considered to have low malignant potential and/or indolent behavior.

There are major differences in incidence, tumor behavior (low versus high malignant potential), and clinical outcome between each histologic subtype. For example, it has been estimated that ~50% of malignant ovarian tumors are serous carcinomas, while ~25% are endometrioid carcinomas, ~10% are mucinous carcinomas, and only ~5% are clear cell carcinomas [45]. However, a study by Seidman et al. reported incidences of 70%, 7%, 10%, and <3% for serous, endometrioid, clear cell, and mucinous carcinomas, respectively, in a series of 220 consecutive cases of invasive ovarian cancer, suggesting that traditional distribution figures may vary considerably [49]. In terms of behavior, serous carcinomas tend to be aggressive, high-grade neoplasms that spread rapidly throughout the pelvis, while endometrioid and mucinous carcinomas are typically low-grade lesions, confined to the ovary [50, 51]. Clear cell and endometrioid carcinomas, unlike other subtypes, are strongly linked to endometriosis [52–56], leading some to believe that endometriosis may be a precursor to these lesions [57, 58]. Tumor histology can also strongly impact clinical response. For instance, late-stage serous and clear cell carcinomas, both of which have similar 5 years survival rates (20–30%), differ markedly in their response to chemotherapy; serous tumors are (initially) highly responsive, while clear cell tumors are notoriously resistant [59, 60]. 

Genetic and biomarker profiling studies of ovarian cancer have revealed that each tumor subtype is associated with a unique “molecular signature.” For example, gene expression profiling can readily distinguish mucinous and clear cell ovarian tumors from other subtypes, regardless of tumor stage or grade [61, 62]. Similarly, microRNA (miRNA) profiling of ovarian carcinomas has identified certain miRNAs that exhibit histotype specificity [63]. Gene expression profiling has also demonstrated that ovarian clear cell tumors are distinctly different from other forms of ovarian cancer and have more in common with clear cell tumors of other organs, such as renal clear cell carcinomas [64]. Even within one histotype, differences in tumor behavior may be underscored by distinct expression profiles. For example, Bonome et al. found that serous borderline tumors (SBTs), which account for 10–15% of serous ovarian neoplasms and are associated with vastly improved survival, cluster separately from high-grade serous carcinomas in hierarchical clustering analyses [65]. Moreover, SBTs are genetically more similar to normal ovarian surface epithelium than to advanced serous tumors. A recent immunohistochemical biomarker study of 500 ovarian carcinomas found that 20 of 21 candidate tumor biomarkers had significantly different expression patterns in each tumor subtype and that two thirds of the biomarkers lost their prognostic value when survival analyses were made subtype specific [66]. These results clearly indicate that each ovarian tumor subtype constitutes a distinct disease and should be treated as such in the contexts of detection, treatment, and prognosis.

 Perhaps the most important characteristic of any tumor is the combination of genetic alterations that underlie its development and drive its progression. In this arena, ovarian tumors again exhibit heterogeneity. Mucinous, endometrioid, and low-grade serous tumors typically acquire mutations in a variety of genes such as *KRAS, BRAF, PTEN*, *β*-*catenin*, and *TFG*-*βRII* [51], all of which belong to signaling pathways controlling cell growth and proliferation, among other processes. Conversely, high-grade serous tumors appear to arise following a mutation in *TP53* [67] or, in the case of familial ovarian carcinoma, *BRCA1, BRCA2, MLH1*, or *MSH2* [68, 69]. All five genes are tumor suppressors that function in DNA damage signaling and repair [69, 70], suggesting that DNA damage is an especially important factor in the etiology of serous ovarian carcinoma. Mutations in *TP53* can even prompt an immunological response, leading to *p53* autoantibody production in some patients with high-grade ovarian carcinomas [71].

Despite the high degree of phenotypic and genotypic variability that exists between different forms of ovarian carcinoma, virtually all patients are treated identically upon diagnosis: cytoreductive surgery, followed by platinum-based chemotherapy. Although many tumors are initially responsive to this treatment, most develop platinum resistance and ~70% recur at some point [72]. Ultimately, only 30% of advanced-stage patients survive five years beyond their diagnoses [1]. Over the past several decades, great advances have been made in the surgical techniques and chemotherapy regimens used to treat ovarian cancer. Yet, despite the best efforts of surgeons, oncologists, and researchers, the 5 years survival rate has improved by only 8% since 1975 [1]. It has thus become apparent that a “blanket approach” to ovarian cancer treatment does not suffice. We must now shift our focus towards the development of targeted therapies capable of exploiting the molecular and genetic characteristics of individual tumor subtypes. This task is made difficult by the fact that we are still very much in the dark when it comes to understanding ovarian cancer etiology. Unlike other malignancies such as cervical or colon cancer, whose pathogeneses are well characterized, the sequence of events leading to ovarian carcinoma development remains a subject of ongoing debate.

## 4. Evolution of the Ovarian Cancer Model

### 4.1. Ovarian Surface Epithelium and Cortical Inclusion Cysts

The traditional view of ovarian cancer asserts that all tumor subtypes share a common origin in ovarian surface epithelium (OSE). OSE is a flat-to-cuboidal layer of uncommitted mesothelial cells covering the exterior surface of the ovary. During ovulation, follicular rupture and oocyte release inflict physical trauma upon the ovarian surface, creating a breach in the OSE that must be repaired. Over the course of a woman's reproductive life, this process of damage and repair is repeated multiple times. Accordingly, OSE cells exhibit a high degree of plasticity that facilitates tissue remodeling; they express both epithelial and mesenchymal markers and can transition from an epithelial to mesenchymal phenotype [73–76]. In addition to physical trauma, OSE cells are subjected to ovulation-associated inflammatory cytokines and reactive oxygen species that are capable of damaging DNA [77]. Accrual of DNA damage by OSE cells may increase their susceptibility to transformation. Furthermore, as women age, the ovarian surface develops numerous invaginations into the cortical stroma. These invaginations frequently pinch off and become entrapped within the stroma, forming circular OSE-lined structures termed “cortical inclusion cysts” (CICs) [78]. Once inside the ovary, the epithelial cells lining CICs are exposed to a new hormone-rich milieu that is thought to induce a differentiation or “metaplasia” into more complex epithelium resembling that of Müllerian-derived organs [78, 79]. Alternatively, it is postulated that in women experiencing endometriosis or endosalpingiosis (i.e., abnormal shedding of endometrial or tubal mucosa, respectively, into the pelvis), remnants of Müllerian-derived epithelia may adhere to the ovarian surface and become incorporated into a CIC [78, 80]. Several hormones acting upon the ovary (e.g., gonadotropins, estrogens, and androgens) have growth-promoting properties that may induce proliferation of epithelial cells within CICs [81]. If the epithelial cells also happen to harbor unresolved DNA damage, they may be prime targets for neoplastic transformation, eventually giving rise to ovarian carcinomas. The OSE-CIC model can account for several important features of ovarian tumorigenesis, including (1) acquisition of Müllerian characteristics by OSE-derived tumors, (2) the cystic nature of benign ovarian tumors and the retention of cystic features by their malignant counterparts, and (3) the presence of low-grade and borderline tumors within the cortical stroma of the ovary. This model is also consistent with well-established epidemiologic data indicating that a decrease in ovulatory cycles (most commonly due to parity or oral contraceptive use) is the greatest risk-reducing factor for ovarian cancer in female populations [82]. However, this model has its limitations. For example, it does not explain why invasive endometrioid and mucinous carcinomas are frequently associated with borderline tumors in the ovary, whereas invasive serous carcinomas are not. Nor does this model address the clear differences in genetic alterations that exist between tumor subtypes. If all ovarian tumors develop from CICs within the ovary, then why do they have such different outcomes and such divergent genotypes? Perhaps the most curious phenomenon, not accounted for by this model, is the existence of extraovarian peritoneal carcinomas. Such tumors are histologically identical to serous ovarian carcinomas but do not involve the ovary and thus, are considered to arise de novo in the peritoneum.

### 4.2. Two-Pathway Model

The two-pathway model was proposed by Shih and Kurman in 2004 in an attempt to integrate most of the clinical, histopathological, and molecular genetic findings concerning ovarian cancer. In particular, they sought to account for the differences in *TP53* and *KRAS* mutational frequencies observed between serous borderline tumors (SBTs) and serous carcinomas [83–85]. SBTs comprise a subset of serous ovarian tumors (including those referred to as atypical proliferative serous tumors and micropapillary serous carcinomas) that are noninvasive, appear to develop from benign serous cystadenomas, and progress very slowly towards low-grade serous carcinoma [86, 87]. The indolent behavior of SBTs contrasts sharply with that of conventional high-grade serous tumors, which spread rapidly and metastasize early in their course. Furthermore, SBTs do not harbor the *TP53* mutations that are characteristic of high-grade serous carcinomas. These observations prompted the formulation of a new model that classifies all ovarian tumors as either Type I or Type II [51]. 

Type I tumors include all major histotypes (serous, endometrioid, mucinous, clear cell, and transitional) but exhibit low-grade nuclear and architectural features, slow growth, and can be linked to well-defined benign ovarian precursor lesions. The most common genetic alterations seen among Type I tumors are *KRAS* and *BRAF* mutations, both of which activate the oncogenic *MAPK* signaling pathway [88]. Mutually exclusive *KRAS* and *BRAF* mutations are observed in ~65% of SBTs but are rarely seen in high-grade serous carcinomas [58, 89]. *KRAS* mutations also occur in ~60% of mucinous, 5–16% of clear cell, and 4-5% of endometrioid Type I carcinomas [51]. *PTEN* mutations, which typically result in constitutive *PI3K* signaling, occur in ~20% of Type I endometrioid neoplasms [90]. The *MAPK* and *PI3K* pathways are related; they eventually converge upon a common downstream translation factor, eIF4B [91], which may represent an important signaling axis in Type I tumor development. *WNT* and *TGF*-*β* signaling pathways are also of potential importance for Type I tumor pathogenesis, based on the presence of *β*-*catenin* mutations in 16–54% of endometrioid tumors and *TGF*-*β* RII mutations in 66% of Type I clear cell tumors [51]. Interestingly, all of the genes altered in Type I ovarian tumors are components of pathways that become intimately related during the process of epithelial-to-mesenchymal transition [92, 93].

Type II ovarian tumors, on the other hand, are infrequently associated with benign or borderline ovarian precursor lesions. They are comprised almost exclusively of high-grade serous carcinomas but also include two less common subtypes—mixed epithelial and undifferentiated carcinomas. Type II ovarian tumors are overwhelmingly *TP53* mutated (50–80%) and may also exhibit gene amplification and overexpression of *HER2/neu* (10–20%) and *AKT2* (12–18%) oncogenes [94–99]. Shih and Kurman's two-pathway hypothesis reconciles most of the phenotypic and genotypic observations pertaining to ovarian tumors and it certainly improves upon the conventional OSE-CIC model. However, their model leaves one critical question unanswered—how do Type II tumors arise and does their pathogenesis include a well-defined precursor lesion? 

### 4.3. Fallopian Tube as a Site of Origin

This question may have been answered by a series of studies investigating the prevalence of occult fallopian tube cancer in women with germline *BRCA* gene mutations. Inherited mutations in *BRCA*1 or *BRCA*2 are associated with familial ovarian and breast cancer syndromes and account for ~11–15% of ovarian carcinomas [100]. Mutations in either gene confer a 15–40% lifetime risk of developing ovarian cancer [101]. Many women with germline *BRCA* mutations (*BRCA*+) elect to undergo risk-reducing bilateral salpingo-oophorectomy (ovary and fallopian tube removal), after which their ovaries are thoroughly examined for evidence of occult cancer. Until recently, the fallopian tubes were not closely examined following such surgeries, and thus, early-stage tubal cancers were rarely detected and severely under reported in *BRCA*+ patients. 

In 2001, Piek et al. drew attention to this issue when they reported that sectioning and examination of fallopian tubes from 12 *BRCA*+ patients revealed a high incidence (50%) of epithelial dysplasia [102]. The fallopian tube epithelium (FTE) is a columnar cell layer composed of two specialized cell types—secretory and ciliated cells. In their study, Piek et al. noticed that dysplastic regions of FTE exhibited a shift towards the secretory phenotype, with complete loss of ciliated cells and the acquisition of proliferative capacity (indicated by Ki67 immunoreactivity). Shortly thereafter, several other groups reported finding occult tubal cancers in the fallopian tubes of *BRCA*+ women, with incidence rates ranging from 0.9–17% [103–105]. 

However, not until a few years later did it become apparent that the distal end of the fallopian tube, the “fimbria”, rather than the proximal region of the tube, was the most crucial site to look for early serous tumors. In 2006, Medeiros et al. conducted a study similar to that of Piek et al., examining the fallopian tubes of 13 *BRCA*+ women undergoing bilateral salpingo-oophorectomy [106]. Medeiros et al. employed a specific protocol for Sectioning and Extensively Examining the FIMbria (SEE-FIM). Their examination uncovered a high incidence (38%) of serous tubal intraepithelial carcinomas in the fallopian tube, but none in the ovaries. Furthermore, 80% of these carcinomas appeared exclusively in the fimbriated end of the fallopian tube, indicating that the fimbria is the preferred site of serous carcinogenesis in *BRCA*+ women. Similarly, Callahan et al. reported on a cohort of 122 *BRCA*+ women in which 7 early carcinomas were found, all originating in the fimbrial/ampullary region. A third study by Kindelberger et al. documented the occurrence of tubal intraepithelial carcinomas (TICs) in 55 consecutive cases of pelvic (i.e., ovarian, tubal, and peritoneal) serous cancer, not selected for *BRCA* status [107]. Of 42 cases designated “serous ovarian carcinoma,” 71% involved the fallopian tube, and 48% of these contained a TIC. Again, TICs were located predominantly (93%) in the fimbrial region. These results suggest that many high-grade serous “ovarian” tumors may actually be of tubal origin, arising from the distal region of the fallopian tube, but then quickly spreading to the nearby ovary.

This concept, while at first provocative, is quite plausible upon consideration. The fimbria lies in extremely close proximity to ovarian surface epithelium and is therefore exposed to the same inflammatory (and potentially carcinogenic) microenvironment. At the fimbriated end of the tube the internal tubal mucosa (endosalpinx) meets the outer tubal serosa which, in turn, is continuous and indistinguishable from the peritoneum. It is therefore easy to imagine that transformed FTE cells, early in their progression, may slough off and migrate to the ovarian surface or directly to the peritoneum, with minimal ovarian involvement. This could explain the existence of some extraovarian peritoneal carcinomas.

**Figure 2:**
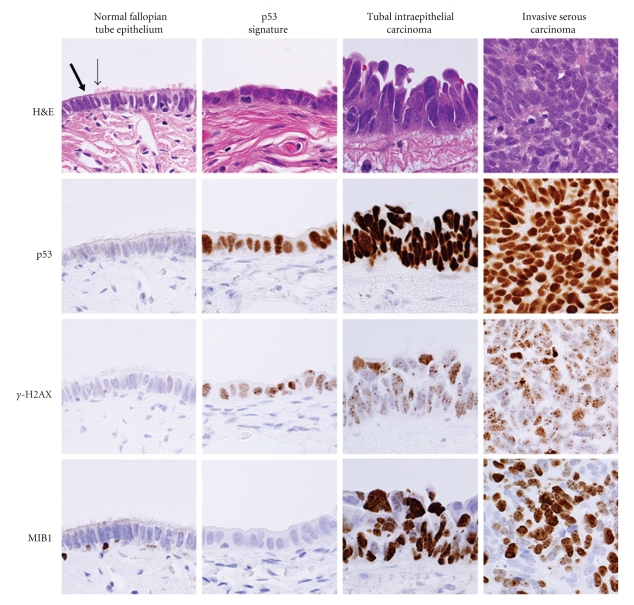
Pathologic features of the morphological continuum from normal fallopian tube epithelium to invasive serous carcinoma. Normal fallopian tube epithelium (FTE), containing both secretory (thick arrow) and ciliated (thin arrow) cells, is typically immunonegative for *p53*, *γ*-H2AX (a marker of DNA damage), and MIB1 (antibody against Ki67; a proliferation marker). The benign “*p53* signature” is composed of a stretch of secretory cells exhibiting strong *p53* expression and evidence of DNA damage (i.e., nuclear *γ*-H2AX foci), but showing no signs of proliferation. Upon progression to TIC, there is an acquisition of proliferative capacity, as evidenced by gain of MIB1 immunoreactivity. High levels of *p53*, *γ*-H2AX, and MIB1 typically persist after a TIC develops into invasive serous carcinoma.

During their initial study of tubal mucosa [106], Medeiros et al. noted stretches of secretory-type cells exhibiting strong *p53* immunoreactivity but appearing nonproliferative and histologically benign. The observed loss of ciliated cells was reminiscent of dysplastic lesions earlier described by Piek et al. [102]. They termed these regions of strong *p53* immunoreactivity “*p53* signatures” (Figure 2). In an attempt to further characterize these entities, Lee et al. examined the occurrence of *p53* signatures in the fimbria of both *BRCA*+ women and women undergoing hysterectomies for benign indications (such as fibroids, endometriosis, or prolapse) [108]. They found that *p53* signatures were equally common in the nonneoplastic fimbria of both *BRCA*+ and control subjects, suggesting that they are a “normal” phenomenon. They also discovered that *p53* signatures stain strongly for the DNA damage marker *γ*-H2AX (Figure 2). Gamma-H2AX is a phosphorylated form of the histone protein H2AX. Phosphorylation of H2AX by DNA damage-sensing kinases ATM and ATR occurs rapidly at sites of DNA double strand breakage [109]. The presence of so-called “*p53* signatures” in the fimbriae of normal women provides the first evidence that, under normal physiologic conditions, fimbrial epithelial cells experience genotoxic damage and trigger a DNA damage response. Lee et al.'s study also made several key observations about the relationship between *p53* signatures and TICs: 1 *p53* signatures occur more frequently in fimbriae where TICs are also present, 2 *p53* signatures are composed exclusively of secretory cells which, like TICs, exhibit a serous phenotype, 3 *p53* signatures and TICs, when concurrent, share evidence of DNA damage and exhibit identical *TP53* mutations, indicating a common origin, and 4 TICs can be distinguished from *p53* signatures by their increased proliferative capacity (i.e., MIB1 positivity and increased Cyclin E expression). Based on their observations, Lee et al. hypothesized that *p53* signatures could represent the elusive ovarian serous carcinoma precursor. To determine whether *p53* signatures also occur in ovarian epithelium, Folkins et al. examined the ovaries and fallopian tubes of 75 *BRCA*+ women. They detected a total of 29 signatures in tubal mucosa but only 1 in OSE and 0 in CICs [110], confirming that *p53* signatures preferentially arise in FTE rather than OSE.

**Figure 3:**
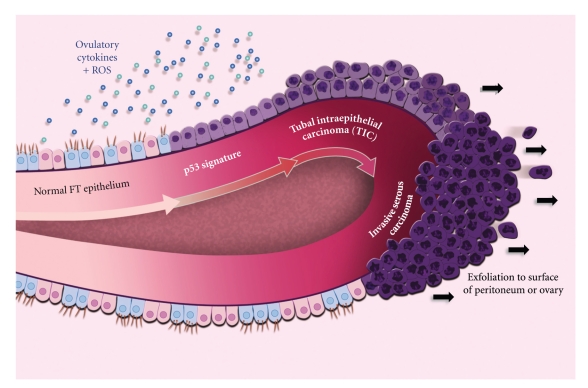
Diagram of a fimbrial plica, illustrating the stepwise progression of normal fallopian tube epithelium to invasive serous carcinoma. The fallopian tube epithelium (FTE) is composed of a single layer of ciliated and secretory cells that are exposed to ovulation-associated inflammatory cytokines and reactive oxygen species (ROS). Repetitive genotoxic stress causes DNA damage and induces *p53* mutation, leading to the clonal expansion of normal looking FTE cells of secretory phenotype. This stretch of damaged cells—termed a “*p53* signature”—stains strongly for *p53* and *γ*-H2AX. Further genetic “hits” enable cells to acquire a proliferative capacity, giving rise to tubal intraepithelial carcinoma (TIC). As a TIC progresses to invasive serous carcinoma, malignant cells are exfoliated from the fimbria, whereupon they may spread rapidly to the surface of the peritoneum and/or ovary. Exfoliation may also occur from TICs prior to fimbrial invasion.

In light of these developments, Lee et al. formulated a model of ovarian cancer which incorporates the fimbria as a major site of origin for serous carcinomas. Their model asserts that there are two distinct pathways leading to ovarian tumorigenesis. The first route is the traditional OSE-CIC pathway, in which OSE (or in some cases FTE, endometrium, or peritoneum) is entrapped within CICs and induced to undergo Müllerian metaplasia within the ovarian stroma, giving rise to mostly endometrioid, mucinous, and serous borderline tumors via a series of step-wise mutations (reviewed in [111]). This pathway leads to the formation of Shih and Kurman's “Type I” tumors. The second pathway involves the fallopian tube fimbria, where a combination of *TP53* mutation and genotoxic stress leads to the clonal expansion of secretory epithelial cells, forming a pre-neoplastic precursor lesion or “*p53* signature.” Additional genetic “hits” in the absence of functional *TP53* enable these cells to acquire a proliferative capacity, facilitating progression to TIC (Figure 3). Identifying the genetic targets of such “hits” is currently a subject of intense research. Serous TICs have the ability to spread rapidly, moving from the fimbria to adjacent pelvic structures (e.g., the ovarian surface, uterine serosa, or peritoneal membranes) or exfoliating into the peritoneal cavity. This second pathway leads to the formation of Shih and Kurman's “Type II” tumors and, importantly, defines a precursor lesion for these tumors. The origin of genotoxic stress in the fimbrial microenvironment remains speculative at this point but is thought to include inflammatory cytokines and reactive oxygen species associated with ovulation. Accordingly, a recent paper has reported that *p53* signatures are associated with lower parity in *BRCA*+ women, suggesting that ovulation is indeed a risk factor for *p53* signature development in the fimbria [112].

This new model of ovarian cancer accounts for nearly all aspects of the disease and, for the first time, describes a step-by-step pathogenesis model for the deadliest and most enigmatic of all ovarian tumors—serous carcinoma. The clinical implications of this model were recently reviewed [111, 113] and suggest that a thorough examination of the fallopian tube fimbria should be conducted during routine pathologic evaluations of salpingo-oophorectomy specimens. It is important to note, however, that this new model is based largely upon descriptive pathological evidence and has not been experimentally validated.

## 5. Experimental Models of Ovarian Cancer

In order to elucidate the molecular mechanisms driving ovarian carcinoma development, we must first be able to model the disease using suitable in vitro and in vivo systems. Several sophisticated mouse models of ovarian carcinoma currently exist. Most are based on the traditional OSE-CIC model of ovarian tumorigenesis and therefore seek to transform murine ovarian surface epithelium (MOSE) in vivo. For example, Connolly et al. induced MOSE transformation in vivo by introducing SV40 T-Ag (large and small) into these cells under the control of a Müllerian inhibitory substance II receptor (MISIIR) promoter element [114]. Other groups have used the Cre-Lox system to conditionally knockout tumor suppressor genes *TP53* and *Rb* in murine ovaries [115]. Orsulic et al. developed a unique model in which a series of oncogenes was introduced directly into the MOSE cells of an adult mouse [116]. Their system employed an avian retroviral vector that requires retroviral receptor (TVA) expression by target cells. This is normally achieved by breeding transgenic animals that express TVA under the control of a tissue-specific gene promoter. However, since no MOSE-specific promoters exist, they removed the ovaries from a TVA expressing mouse, infected them in vitro, then implanted them orthotopically into a recipient mouse. Using this method, they were able to demonstrate the importance of *TP53* in suppressing ovarian tumorigenesis. Specifically, they showed that introduction of a trio of oncogenes (*c-MYC*, *KRAS*, and *AKT*) into *p*53^+/+^ MOSE could not induce tumor formation, while introducing any two of the same oncogenes induced rapid tumor formation in *p*53^−/−^ mice. All three of these models gave rise to poorly differentiated tumors resembling serous ovarian carcinomas and exhibited intraperitoneal dissemination.

 There have also been attempts to create subtype-specific mouse models. Dinulescu et al. successfully generated tumors with endometrioid histology by using the Cre-Lox system to conditionally activate oncogenic *KRAS* and inactivate *PTEN* [117]. Using this system, they demonstrated that expression of activated *KRAS* or deletion of *PTEN* in MOSE led to an endometriosis-like condition, characterized by the presence of benign glandular lesions in the ovary. In contrast, the combination of both genetic mutations induced formation of invasive and metastatic adenocarcinomas resembling human ovarian endometrioid tumors. Their model nicely recapitulates the stepwise progression of Type I ovarian tumors. However, *KRAS* mutations are not commonly found in the endometrioid subtype. A second endometrioid tumor model developed by Wu et al. used the Cre-Lox system to delete *PTEN* and *APC* tumor suppressor genes, again giving rise to tumors with endometrioid histologies [118]. Although *APC* mutations are not characteristic of endometrioid ovarian carcinomas, they serve to deregulate the same pathway as *β*-*catenin* mutations—specifically, the *WNT* signaling pathway. 

Mouse models that can faithfully reproduce both the genotypic and phenotypic characteristics of ovarian tumors are crucial for the development of targeted therapies. Current ovarian carcinoma models indicate that there are two divergent pathways to ovarian tumorigenesis, each giving rise to tumors with very different characteristics (Types I and II). In attempting to model these tumor types, it is important to consider that many Type II tumors may arise from extraovarian sites—most notably, the fallopian tube epithelium. This is of critical importance, for example, in the context of orthotopic tumor cell implantation.

Recent revelations regarding the tubal origin of high-grade serous ovarian carcinomas present a challenge because, while human OSE cells have been routinely cultured for many years, FTE cells are a relatively scare commodity. There are no readily available human FTE cell lines and primary human FTE can be difficult to obtain. Nevertheless, models of FTE must be created in order to study the mechanisms of tubal/ovarian serous carcinoma development. Only a handful of labs thus far have attempted to culture or propagate FTE cells in vitro, the majority of them working within the field of reproductive biology [119–124]. As mentioned earlier, the FTE is a complex columnar epithelium composed of both secretory and ciliated cell types. However, ciliated FTE cells tend to either die or rapidly dedifferentiate when grown in vitro, resulting in a complete loss of the ciliated cell phenotype from FTE cocultures. Serous tubal/ovarian carcinomas are exclusively secretory in nature, suggesting that ciliated cells must be either less susceptible to malignant transformation or more apt to undergo cell death in response to genotoxic stressors in their microenvironment. To address such issues, a model system is required in which both ciliated and secretory cells remain viable, so that the behaviors of each cell type may be readily compared and contrasted. 

To preserve the phenotypic integrity of FTE cocultures, they may be grown on collagen-coated porous filters, as described by Rajagopal et al. for the culture of monkey oviductal cells [125]. Similar techniques have been used to construct polarized cultures of respiratory epithelial cells [126]. Our lab has recently established an analogous “ex vivo” model of human FTE (unpublished data). Using this FTE coculture system we aim to answer several important questions, including the following: What are the molecular mechanisms driving FTE malignant transformation? Can transformed FTE cells progress to serous tubal intraepithelial carcinoma? Which molecules may be targeted in a therapeutic context to halt serous carcinoma progression? Are there FTE-specific genes that can be exploited for the development of a tubal serous carcinoma mouse model? Can we identify novel biomarkers relevant to serous tumor progression? Our studies must ultimately also consider the role of the tumor microenvironment, since it is apparent that multicellular interactions contribute to serous tumor metastasis and are important factors in a therapeutic context [127, 128]. Overall, these research efforts will offer new insights into the nature of the FTE and its propensity for malignant transformation, and thus, advance our understanding of ovarian cancer pathogenesis.
